# Genetic characterization of ESBL-producing and ciprofloxacin-resistant *Escherichia coli* from Belgian broilers and pigs

**DOI:** 10.3389/fmicb.2023.1150470

**Published:** 2023-04-06

**Authors:** Sien De Koster, Moniek Ringenier, Basil Britto Xavier, Christine Lammens, Dieter De Coninck, Katrien De Bruyne, Klaas Mensaert, Marjolein Kluytmans-van den Bergh, Jan Kluytmans, Jeroen Dewulf, Herman Goossens, on behalf of the i-4-1-Health Study Group

**Affiliations:** ^1^Laboratory of Medical Microbiology, Vaccine and Infectious Diseases Institute, University of Antwerp, Antwerp, Belgium; ^2^Veterinary Epidemiology Unit, Department of Reproduction, Obstetrics and Herd Health, Faculty of Veterinary Medicine, Ghent University, Merelbeke, Belgium; ^3^HIV/STI Unit, Department of Clinical Sciences, Institute of Tropical Medicine, Antwerp, Belgium; ^4^Hospital Outbreak Support Team-HOST, ZNA Middelheim, Antwerp, Belgium; ^5^Hospital Outbreak Support Team-HOST, GZA Ziekenhuizen, Wilrijk, Belgium; ^6^BioMérieux SA, Sint-Martens-Latem, Belgium; ^7^Department of Infection Control, Amphia Hospital, Breda, Netherlands; ^8^Julius Center for Health Sciences and Primary Care, UMC Utrecht, University of Utrecht, CG Utrecht, Netherlands; ^9^Amphia Academy Infectious Disease Foundation, Amphia Hospital, CK Breda, Netherlands; ^10^Microvida Laboratory for Microbiology, Amphia Hospital, Breda, Netherlands

**Keywords:** ESBL, ciprofloxacin, *Escherichia coli*, livestock, WGS

## Abstract

**Background:**

The increasing number of infections caused by *Escherichia coli* resistant to clinically important antibiotics is a global concern for human and animal health. High overall levels of extended-spectrum beta-lactamase (ESBL)-producing and ciprofloxacin-resistant (ciproR) *Escherichia coli* in livestock are reported in Belgium. This cross-sectional study aimed to genotypically characterize and trace ESBL-and ciproR-*E. coli* of Belgian food-producing animals.

**Methods:**

A total of 798 fecal samples were collected in a stratified-random sampling design from Belgian broilers and sows. Consequently, 77 ESBL-*E. coli* and 84 ciproR-*E. coli* were sequenced using Illumina MiSeq. Minimum inhibitory concentration (MIC) for fluoroquinolones and cephalosporins were determined. Molecular *in silico* typing, resistance and virulence gene determination, and plasmid identification was performed. Scaffolds harboring ESBL or plasmid-mediated quinolone resistance (PMQR) genes were analyzed to detect mobile genetic elements (MGEs) and plasmid origins. Core genome allelic distances were used to determine genetic relationships among isolates.

**Results:**

A variety of *E. coli* sequence types (ST) (*n* = 63), resistance genes and virulence profiles was detected. ST10 was the most frequently encountered ST (8.1%, *n* = 13). The pandemic multidrug-resistant clone ST131 was not detected. Most farms harbored more than one ESBL type, with *bla*_CTX-M-1_ (41.6% of ESBL-*E. coli*) being the most prevalent and *bla*_CTX M-15_ (*n* = 3) being the least prevalent. PMQR genes (15.5%, *n* = 13) played a limited role in the occurrence of ciproR-*E. coli*. More importantly, sequential acquisition of mutations in quinolone resistance-determining regions (QRDR) of *gyrA* and *parC* led to increasing MICs for fluoroquinolones. GyrA S83L, D87N and ParC S80I mutations were strongly associated with high-level fluoroquinolone resistance. Genetically related isolates identified within the farms or among different farms highlight transmission of resistant *E. coli* or the presence of a common reservoir. IncI1-I(alpha) replicon type plasmids carried different ESBL genes (*bla*_CTX-M-1_, *bla*_CTX-M-32_ and *bla*_TEM-52C_). In addition, the detection of plasmid replicons with associated insertion sequence (IS) elements and ESBL/PMQR genes in different farms and among several STs (e.g., IncI1-I(alpha)/IncX3) underline that plasmid transmission could be another important contributor to transmission of resistance in these farms.

**Conclusion:**

Our findings reveal a multifaceted narrative of transmission pathways. These findings could be relevant in understanding and battling the problem of antibiotic resistance in farms.

## 1. Introduction

*Escherichia coli* remains one of the most important pathogens for humans (Murray et al., 2022), as evidenced by its contribution to mortalities due to drug resistance. Fluoroquinolones and beta-lactam antibiotics are life savers in both human (World Health Organization Advisory Group on Integrated Surveillance of Antimicrobial Resistance, 2018) and animal healthcare (World Organization for Animal Health (OIE), 2018): these medications are essential for treating severe illnesses. Resistance to extended-spectrum cephalosporins and fluoroquinolones constitutes a major public health problem because this limits the treatment options for serious bacterial infections (World Health Organization Advisory Group on Integrated Surveillance of Antimicrobial Resistance, 2018) and drives the use of the last resort of antibiotic therapy, i.e., carbapenems. The gastrointestinal tract of animals serves as a reservoir of antimicrobial resistance (AMR), which can spread *via* MGEs (Moor et al., 2021). The presence of resistance genes on MGEs enables their dispersion, posing a great hazard to food safety (Partridge et al., 2018). Clinically significant ESBL genes, belonging to the *bla*_CTX-M_, *bla*_TEM_ and *bla*_SHV_ gene families, can successfully disseminate because they are commonly located on plasmids (IncA/C, IncF, IncHI1, IncHI2 IncI, IncK, IncN, IncX plasmids) (Rozwandowicz et al., 2018). In addition, three mechanisms of plasmid-mediated quinolone resistance (PMQR) are known: protection of DNA gyrase and topoisomerase IV from quinolone inhibition by *qnr* genes (ColE plasmids) (Tran et al., 2005), acetylation of quinolones by aminoglycoside acetyltransferase Aac(6′)-Ib-cr (Robicsek et al., 2006) and quinolone accumulation due to quinolone efflux pumps QepAB (Yamane et al., 2007) and OqxAB (ColE plasmids, IncX plasmids) (Hansen et al., 2007; Jacoby et al., 2015; Rozwandowicz et al., 2018). These mechanisms provide low-level resistance (ciprofloxacin MIC range: 0.06–0.25 mg/l); however, they are usually present on multidrug-resistant (MDR) plasmids and facilitate the selection of higher-level resistance making infections with PMQR-carrying pathogens harder to treat (Jacoby et al., 2015). Quinolone resistance in Gram-negative bacteria can also be caused by single amino acid changes in QRDRs in DNA gyrase (*gyrA*) and DNA topoisomerase IV (*parC*) (Karczmarczyk et al., 2011; Gordon and George, 2015). Another mechanism contributing to (fluoro)quinolone resistance is the increased expression of the AcrAB-TolC efflux pump which is regulated by repressor AcrR and other regulators of drug efflux MarAR and SoxRS as well as RNA polymerase RpoB (Amábile-cuevas and Demple, 1991; White et al., 1997; Oethinger et al., 1998; Lindgren et al., 2003; Pietsch et al., 2017) and the AcrB component of the efflux pump itself (White et al., 1997; Blair et al., 2015).

A previous study indicated a high occurrence of ESBL-producing and ciprofloxacin-resistant *E. coli* in fecal samples of broilers and pigs in Belgian farms (De Koster et al., 2021). Possible explanations for these observations include the dissemination of resistant *E. coli* vertically along the production chain from one generation to another (Dierikx et al., 2013; Zurfluh et al., 2014) and resistant *E. coli* residing in the farm environment (Blaak et al., 2015) along with the dissemination of resistant *E. coli* or their resistance genes between farm animals (Hayer et al., 2020). However, the research into the genetic diversity and antibiotic resistance of *E. coli* that colonize livestock in Belgian farms has been limited. Most studies of commensal *E. coli* in livestock, such as the Belgian Antimicrobial Consumption and Resistance in Animals (AMCRA) reports (FAVV-AFSCA, 2021), the European Food Safety Authority (EFSA) and European Centre for Disease Prevention and Control (ECDC) reports (European Food Safety Authority and European Centre for Disease Prevention and Control, 2021) rely on phenotypic AMR profiles. The lack of whole genome sequencing (WGS) to track MDR and high-risk clones was acknowledged in the latest BELMAP report, which aims to summarize monitoring programs in Belgium and recommends improving monitoring (FOD Volksgezondheid Veiligheid van de Voedselketen en Leefmilieu et al., 2021). An interdisciplinary One Health strategy is essential for tracking AMR’s spread between humans, animals and their shared environment. Data on *E. coli* found in food-producing animals should be utilized to identify potential pathways of transmission through which the risk may reach human populations through consumption. To investigate the molecular epidemiology of ESBL-*E. coli* and ciproR-*E. coli*, we used WGS to identify resistance genes, mutations and potential transmission pathways between and among farms.

## 2. Materials and methods

### 2.1. Setting, study period and sample/isolate collection

Within the framework of the i-4-1-Health project, a total of 798 fecal samples were collected in a stratified-random sampling design from conventional broiler (*n* = 15) and multiplier sow farms (*n* = 15) in Flanders, Belgium (September 2017–April 2018). When present, sampling was conducted in different units (broiler houses or rooms with weaned pigs) with a maximum of three units per farm. The farms were included based on the relative level of antibiotic use, meaning that antibiotic use was higher than average compared to the national benchmark value in the respective countries. Farm characteristics and antibiotic use were described previously (Caekebeke et al., 2020).

### 2.2. ESBL-producing and ciprofloxacin-resistant *Escherichia coli*

Isolation of ESBL- and ciproR-*E. coli* was performed as described by Kluytmans-van den Bergh et al. (2019). A total of 724 ESBL-*E. coli* and 467 ciproR-*E. coli* were isolated from the fecal samples. To investigate the molecular epidemiology, three ESBL-*E. coli* and three ciproR-*E. coli* from each farm were chosen for in-depth analysis including phenotypic characterization and whole genome sequencing. In particular, the first ESBL-*E. coli* and ciproR-*E. coli* isolated from each farm unit were selected. In farms with one sampled unit, three ESBL-*E. coli* and ciproR-*E. coli* with a distinct antibiotic susceptibility profile were selected from that unit. Using these selection criteria, 82 ESBL-*E. coli* [broiler (*n* = 45), pig (*n* = 37)] and 84 ciproR-*E. coli* [broiler (*n* = 45), pig (*n* = 39)] were selected for MIC determination and whole genome sequencing.

### 2.3. Whole genome sequencing

A single colony was inoculated in 4 ml Mueller Hinton broth and incubated overnight at 35–37°C. The MasterPure Complete DNA & RNA Purification kit (Epicentre, Madison, WI, USA) was used to extract genomic DNA. Libraries were prepared using the Nextera XT sample preparation kit (Illumina, San Diego, CA, USA) and sequenced with 2× 250 bp paired-end sequencing using the Illumina MiSeq platform (Illumina, San Diego, CA, USA). The sequencing data were submitted to NCBI under BioProject PRJNA905236. Supplementary Table 1 provides an overview of ESBL-*E. coli* and ciproR-*E. coli* sequences and their genetic characteristics used in this study.

### 2.4. *De novo* assembly, genotyping and phylogenetic analysis

Sequences were trimmed with TrimGalore v.0.4.4^1^ and assembled *de novo* using SPAdes v.3.13.0 (Bankevich et al., 2012). Assembly quality was assessed with Quast (Gurevich et al., 2013). The assembled genome was annotated using Prokka v.1.12 (Seemann, 2014). Additional analysis was performed using BacPipe v1.2.6 (Xavier et al., 2020) including the PubMLST database (Achtman scheme) (Jolley et al., 2018), the CARD database (McArthur et al., 2013), ResFinder v4.1 (Bortolaia et al., 2020), VirulenceFinder v2.0.3 (Tetzschner and Lund, 2020) and PlasmidFinder v2.0 (Carattoli and Hasman, 2020). Serotype and pathotype were determined using BioNumerics v7.6.3 (Applied Maths NV, Sint-Martens-Latem, Belgium). The identification of pathotypes was performed according to the virulence factor database (VFDB) (Chen et al., 2016). *In silico* prediction of fimH type and H and O serotypes was performed using FimTyper 1.0 (Roer et al., 2017) and SeroTypeFinder (Joensen et al., 2015), respectively. Phylogroups were determined using ClermonTyping (Beghain et al., 2018). For core genome multilocus sequence typing (cgMLST), a gene-by-gene approach was employed by generating a study-specific scheme and analyzing allelic loci distances of cgMLST using ChewBBACA (Silva et al., 2018) and visualizing the tree using iTOL v6 (Letunic and Bork, 2021).

### 2.5. Phenotypic and genotypic antibiotic resistance determination

ESBL production was phenotypically confirmed using the combination disk diffusion method. Ciprofloxacin resistance was confirmed by ciprofloxacin MIC determination using VITEK^®^ MS system (bioMérieux, Marcy l’Etoile, France). In addition, MICs for amoxicillin-clavulanic acid, ampicillin, cefuroxim, cefotaxime, ceftazidime, cefoxitin, fosfomycin, gentamicin, imipenem, meropenem, nitrofurantoin, piperacillin-tazobactam, tobramycin, trimethoprim were determined using VITEK^®^ MS system (bioMérieux, Marcy l’Etoile, France). Furthermore, ciprofloxacin, enrofloxacin, levofloxacin and moxifloxacin were tested for 106 *E. coli* of which 18 were ciprofloxacin-susceptible *E. coli* and 88 were ciprofloxacin non-susceptible *E. coli* using E-tests (bioMérieux, Marcy l’Etoile, France) to identify genome-wide associations between genetic markers and fluoroquinolone resistance levels. Results were interpreted using the EUCAST breakpoint tables v12.0 (The European Committee on Antimicrobial Susceptibility Testing, 2022) and an enrofloxacin breakpoint of MIC≤0.25 mg/l (Hao et al., 2013). After sequencing, known ESBL genes could not be detected in five phenotypic ESBL-*E. coli* (5/82, 6%) (from broiler farms one, four and eight and pig farms three and fifteen); therefore, these isolates were excluded, resulting in 77 ESBL-*E. coli* for further analysis. QRDRs were investigated for mutations conferring resistance within gyrase *gyrA* and *gyrB* and topoisomerases IV *parC* and *parE*. In addition, mutations in *acrB*, *acrR*, *marA*, *marR*, *rpoB*, *soxR*, *soxS* were considered. Mutations and predicted amino acid changes were aligned using clustalw, inbuilt within the CLC genomics workbench v.9.5.3 (CLC bio, Denmark). Prediction of whether amino acid changes affect protein function was performed by Sorting Intolerant From Tolerant (SIFT) (Ng and Henikoff, 2003). Scaffolds containing ESBL or PMQR genes were analyzed using MGEFinder v1.0.3 (Durrant et al., 2020), and ISFinder (Siguier et al., 2006) to detect MGEs and replicon types of plasmids. Scaffolds containing ESBL genes or PMQR represent plasmid sequences were analyzed further on NCBI using blastn search with default settings to the blast database v5. Resistance genes were classified as Rank I (human-associated, mobile ARGs, in ESKAPE pathogens, current threats) or Rank II (human-associated, mobile ARGs emerging from non-pathogens, future threats) (Zhang et al., 2021; Supplementary Table 2).

### 2.6. Statistical tests and visualization

Statistical tests and visualization of the presence of resistance genes, virulence genes and plasmids were performed using R version 4.2.0 (R Core Team, 2020). Differences in the presence of genes were tested using a One-way ANOVA and TukeyHSD test in case of equal variances or a Welch ANOVA and the Games-Howell test in case of unequal variances (mean ± standard deviation and *p*-values are shown). Associations of genetic markers with a phenotype were examined using phi and chi-squared test.

## 3. Results

### 3.1. ESBL and PMQR genes in ESBL-producing and ciprofloxacin-resistant *Escherichia coli*

**Figure 1:**
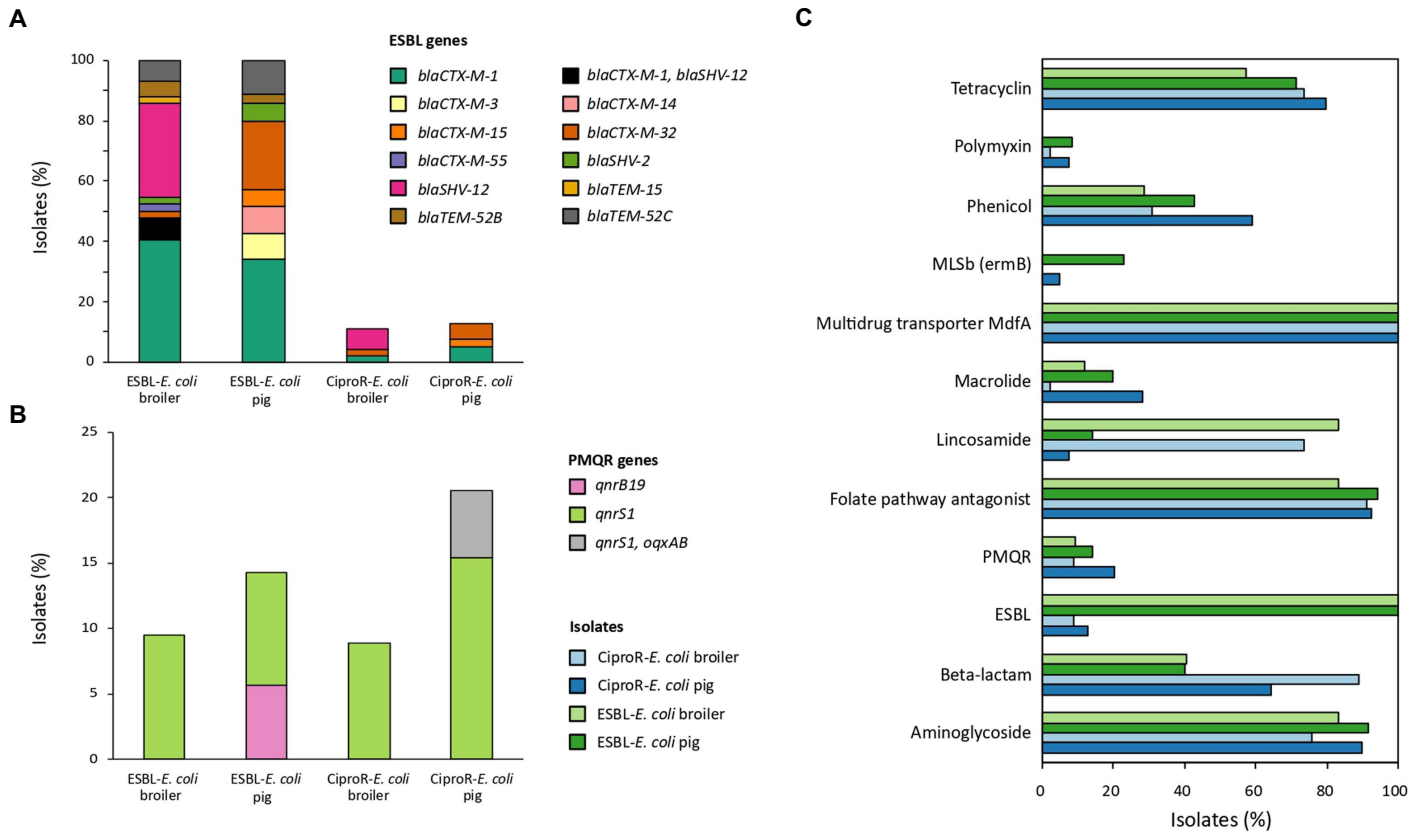
The percentage of isolates carrying ESBL genes **(A)**, plasmid-mediated quinolone resistance (PMQR) genes **(B)** and genes conferring resistance to other antibiotic classes **(C)**.

The most abundant ESBL genes detected in *E. coli* isolated from broilers were *bla*_CTX-M-1_ (40.5%, *n* = 17) followed by *bla*_SHV-12_ (31.0%, *n* = 13). Other ESBL genes detected in broiler isolates were *bla*_CTX-M-32_ (2.4%), *bla*_CTX-M-55_ (2.4%), *bla*_SHV-2_ (2.4%), *bla*_TEM-15_ (2.4%), *bla*_TEM-52B_ (4.8%) and *bla*_TEM-52C_ (7.1%). Three isolates (7.1%) from different broiler farms harbored *bla*_CTX-M-1_ and *bla*_SHV-12_. *Bla*_CTX-M-1_ was also the most common in *E. coli* from pigs (34.3%, *n* = 12), followed by *bla*_CTX-M-32_ (22.9%), *bla*_TEM-52C_ (11.4%), *bla*_CTX-M-3_ (8.6%), *bla*_CTX-M-14_ (8.6%), *bla*_CTX-M-15_ (5.7%), *bla*_SHV-2_ (5.7%), *bla*_TEM-52B_ (2.9%) in pig isolates (Figure 1A). Eight of the ciproR-*E. coli* also harbored *bla*_CTX-M-1_ (*n* = 2), *bla*_CTX-M-32_ (*n* = 2), *bla*_CTX-M-15_ (*n* = 1), *bla*_SHV-12_ (*n* = 2) and one isolate with both *bla*_CTX-M-1_ and *bla*_SHV-12_. PMQR genes were found in a relatively low number of ciproR-isolates (14.3%, *n* = 12) (Figure 1B). Of the 84 ciproR-*E. coli*, 12 isolates harbored *qnrS1* (8.9% of the broiler isolates and 15.4% of the pig isolates). Two pig isolates (5.1%) additionally contained the efflux pump OqxAB. A total of 9.5% of the ESBL-*E. coli* from broilers and 8.6% of the ESBL isolates from pigs harbored *qnrS1*. Also, *qnrB19* was detected in 5.7% of porcine ESBL-*E. coli*.

### 3.2. Other resistance genes, virulence genes and plasmids

In total, 95.8% of the isolates were MDR (i.e., resistant to at least 3 antibiotic classes (Magiorakos et al., 2012)). Genes conferring resistance to aminoglycosides were abundant (overall in 84.5% of the isolates), folate pathway antagonists were present in 90.1% of the isolates, and all isolates harbored multidrug transporter MdfA. Lincosamide resistance was often detected in broiler isolates (ciproR-*E. coli*: 73.3%, ESBL-*E. coli* 83.3%) and beta-lactam resistance was often detected in ciproR-*E. coli* (pig: 64.1%, broiler: 88.9%) (Figure 1C). Plasmid-mediated colistin resistance was found in three pig farms [*mcr-1.1* (*n* = 1), *mcr-2.1* (*n* = 2), *mcr-9* (*n* = 1)] and in one broiler farm [*mcr-9* (*n* = 1)]. Both *mcr-9*- containing isolates did not have the complete *qseC-qseB* two-component system to induce colistin resistance. Highly diverse resistance gene profiles (131 different profiles among 161 isolates) were detected within the same farm and between farms.

**Figure 2:**
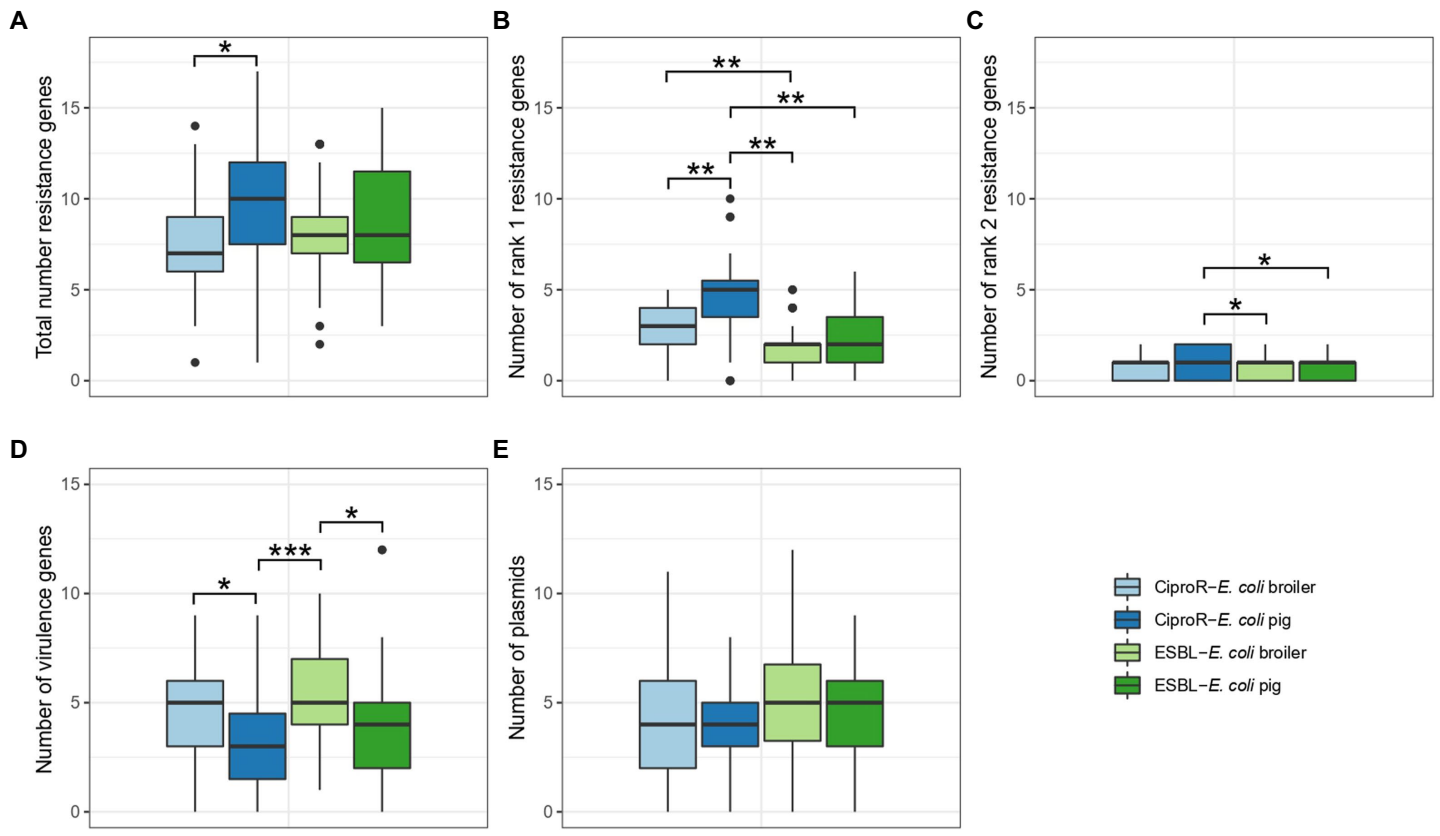
Number of resistance genes **(A–C)**, virulence genes **(D)** and plasmids **(E)** in ESBL-*Escherichia coli* and CiproR-*E. coli* isolated from broilers and pigs. Statistically significant differences are indicated according to the level of significance: * (*p* < 0.05), ** (*p* < 0.01), *** (*p* < 0.001) (ANOVA with TukeyHSD or Games-Howell post-hoc tests).

The mean number of resistance genes was significantly higher (*p* < 0.05) in ciproR-*E. coli* from pigs (9.44 ± 4.01) compared to ciproR-*E. coli* from broilers (7.51 ± 2.85) (Figure 2A). Resistance genes that are a current threat to public health, referred to as Rank I resistance genes, were more abundantly present in ciproR-*E. coli* compared to ESBL-*E. coli* and more in pig isolates (4.6 ± 2.4 Rank I resistance genes) compared to broiler isolates (2.8 ± 1.4 Rank I resistance genes) (*p* < 0.01) (Figure 2B). Similar observations can be made for Rank II resistance genes (considered future threats) which were present in higher numbers in porcine ciproR-*E. coli* compared to ESBL-*E. coli* from both broilers and pigs (*p* < 0.05) (Figure 2C). On the other hand, broiler isolates contain a higher number of virulence genes (ciproR-*E. coli*: 4.62 ± 2.23; ESBL-*E. coli*: 5.45 ± 2.60) compared to pig isolates (ciproR-*E. coli*: 3.10 ± 2.25; ESBL-*E. coli*: 3.97 ± 2.81) (Figure 2D). This divergence of resistance and virulence was observed in the higher number of virulence genes (up to 12 genes) and lower number of Rank I resistance genes in ESBL-*E. coli*, while the opposite was seen for most ciproR-*E. coli*, which can carry a higher number of Rank I resistance genes (up to 10 Rank I resistance genes) (Supplementary Figure 1). Fourteen isolates showed a convergence of virulence and resistance (at least 3 Rank I resistance genes and more than six virulence genes) which belonged to ST117, ST189 (*n* = 2), ST648, ST88, ST1011, ST75, ST624, ST115 (*n* = 3), ST48 and ST350 (*n* = 2). Overall, a large diversity was seen in the number of virulence and Rank I resistance genes ranging from lower-risk (one resistance gene and one virulence gene) to high-risk isolates (five Rank I resistance genes and nine virulence genes) (Supplementary Figure 1). On average, four plasmids were detected per isolate and no significant differences in the number of plasmids between the isolates of different origins were detected (Figure 2E). The most common replicon markers (>10% in one or more categories) were IncFIB (52.9%), IncI1-I (gamma) (38.2%), Col (MG828) (30.1%), IncFII (27.7%), IncX1 (25.6%), IncFIC(FII) (23.6%) and p0111 (18.9%). Plasmid replicon IncB/O/K/Z was exclusively detected in broiler isolates (in 23.0% of ciproR-*E. coli* and in 28.9% of ESBL-*E. coli*) (Supplementary Figure 2). Most virulence genes were involved in adherence and invasion (Supplementary Figure 3). The most prevalent virulence genes were *iss* (75%), *gad* (57%), *lpfA* (37%) and *iroN* (37%). A total of 120 different virulence profiles were detected within farms.

### 3.3. Genotype–phenotype correlations for resistance in ESBL-*Escherichia coli* and ciproR-*Escherichia coli*

More than one type of ESBL gene was detected in most of the sampled farms (73.3%; 22/30 farms). All ESBL genes were associated with very high ampicillin (MIC ≥32 mg/l) and cefotaxime (MIC 8 to ≥64 mg/l) resistance levels (*p* < 0.001), except for two *bla*_SHV-2_-harboring porcine isolates which showed cefotaxime MICs below breakpoint (MIC ≤1 mg/l). Strong levels of agreement between ESBL genotype and phenotype were detected for cefuroxime (89.44%, phi coefficient: 0.76), and ceftazidime (86.96%, phi coefficient: 0.77) and an almost perfect level of agreement was detected for cefotaxime (98.14%, phi coefficient: 0.96) (Table 1).

**Table 1:** Concordance between ESBL genotypes and cephalosporin phenotypes in *Escherichia coli* isolates from livestock.

Antibiotic	Susceptible phenotype		Non-susceptible phenotype		Agreement (%)	Phi coefficient (95% CI)	*p*-value
	ESBL gene presence	ESBL gene absence	ESBL gene presence	ESBL gene absence			
Cefuroxim	7 (4.3%)	66 (41.0%)	78 (48.5%)	10 (6.2%)	89.44	0.76 (0.69–0.88)	***(<0.001)
Cefotaxime	2 (1.2%)	75 (46.6%)	83 (51.6%)	1 (0.6%)	98.14	0.96 (0.91–1)	***(<0.001)
Ceftazidime	19 (11.8%)	75 (46.6%)	65 (40.4%)	1 (0.6%)	86.96	0.77 (0.67–0.87)	***(<0.001)

CI: confidence interval.

**Figure 3:**
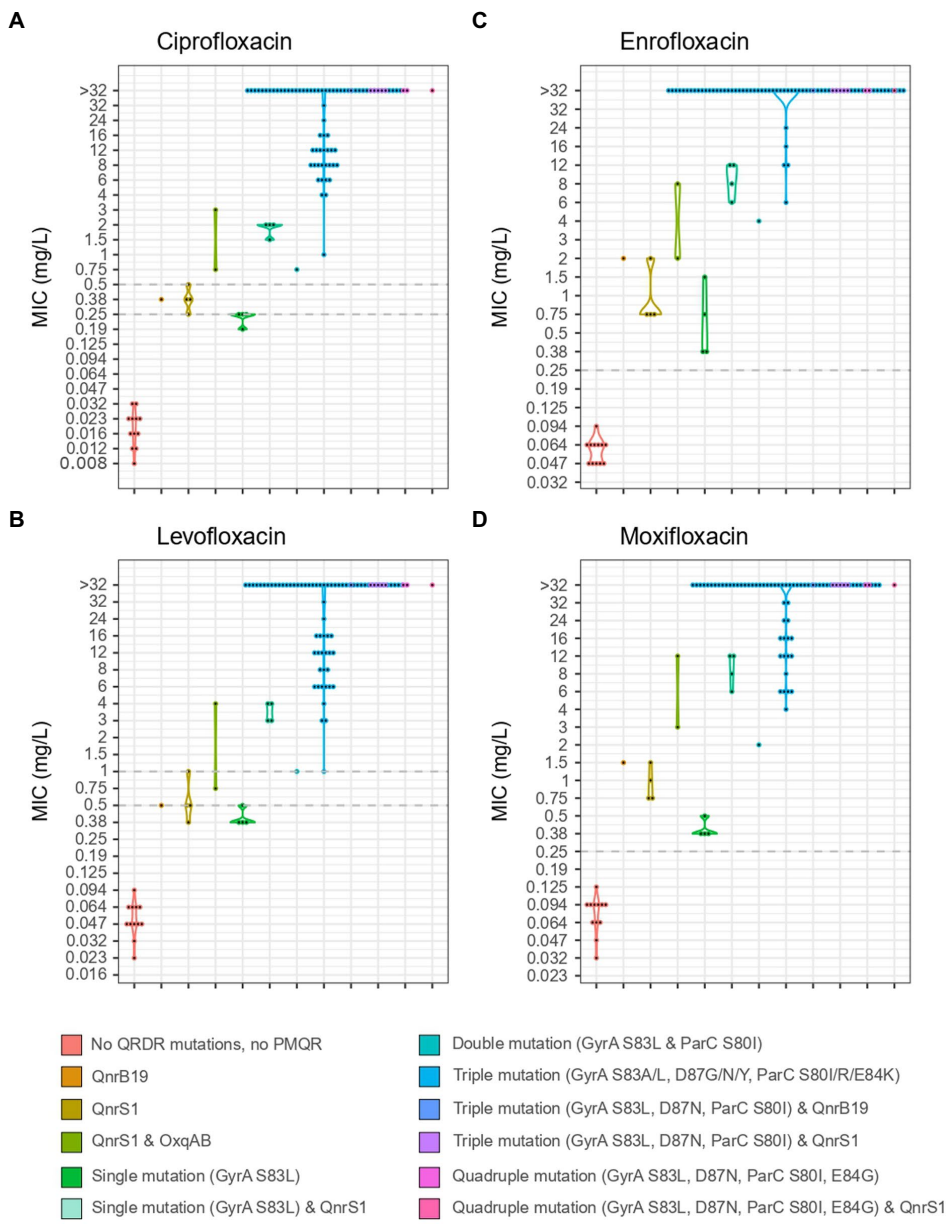
MIC values for ciprofloxacin **(A)**, levofloxacin **(B)**, enrofloxacin **(C)** and moxifloxacin **(D)** of 106 isolates from Belgian broilers and pigs in association with the mutations in QRDR regions of GyrA and ParC and the presence of PMQR genes. EUCAST breakpoints are indicated with a horizontal, dotted grey line.

Mutations in QRDR of *gyrA* and *parC* were found in all ciproR-*E. coli*. Sequential acquisition of individual mutations in QRDR of *gyrA* and *parC* led to increasing MICs for all tested fluoroquinolone antibiotics. Predicted amino acid change S83L in GyrA caused low-level resistance to enrofloxacin and moxifloxacin, but not to ciprofloxacin and levofloxacin. Triple or quadruple mutations in QRDR caused high-level fluoroquinolone resistance (MIC>4 mg/l). QnrS1 or QnrB19 alone leads to low-level resistance to enrofloxacin and moxifloxacin and a sensitive/intermediate phenotype for ciprofloxacin and levofloxacin. The presence of both *oqxAB* and *qnrS1* genes lead to a non-susceptible phenotype for all four fluoroquinolones (Figure 3).

**Figure 4:**
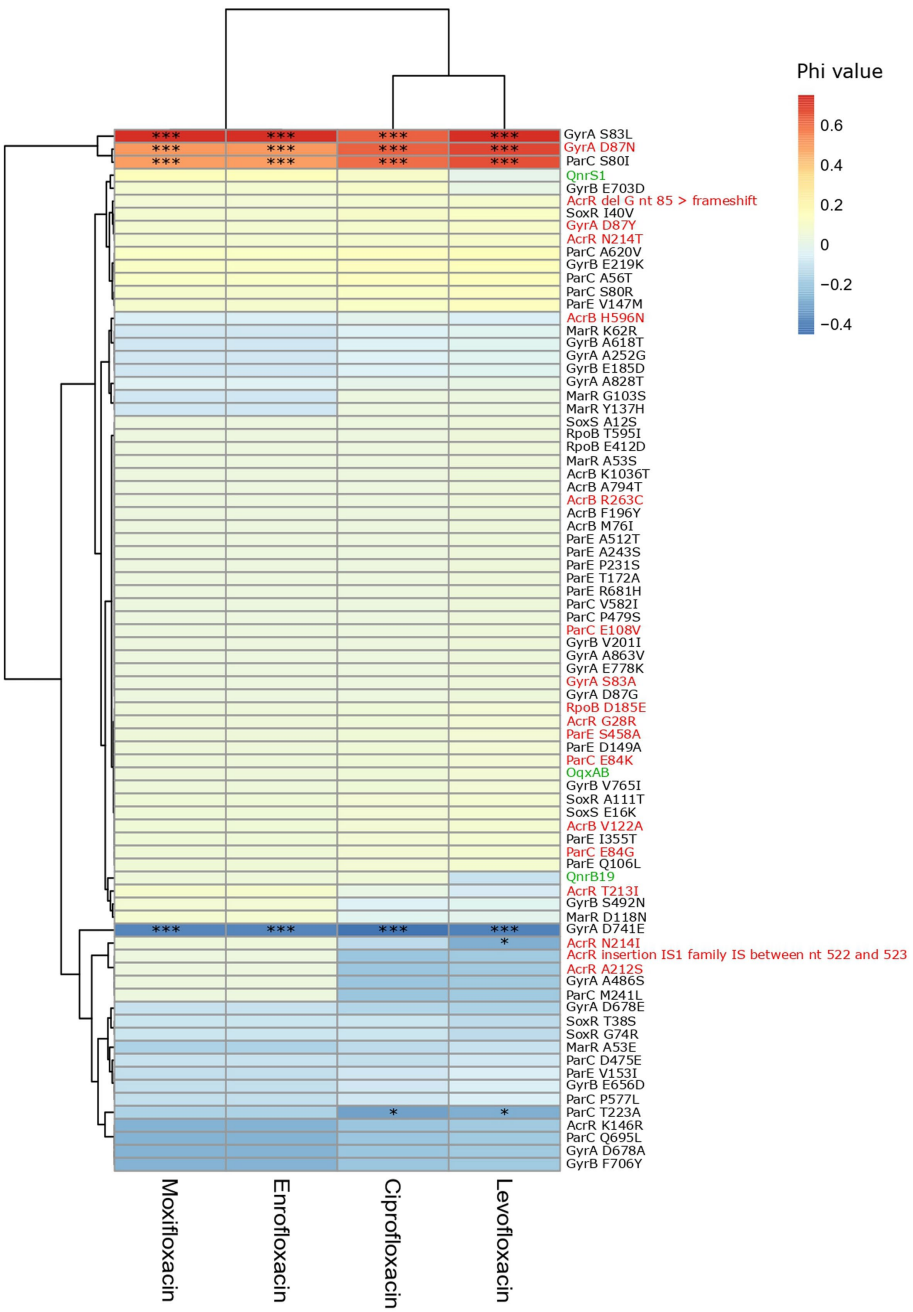
Heatmap of the association between the presence of PMQR genes and mutations and fluoroquinolone non-susceptibility. Colors represent the phi values. Negative phi values represent negative associations, positive values represent positive associations between the genes/mutations and the non-susceptibility to the fluoroquinolone antibiotics. PMQR genes are indicated in green, predicted amino acid changes that are likely deleterious for the protein function according to SIFT are indicated in red. *(*p* < 0.05), *** (*p* < 0.001) (Chi-squared test). IS: insertion sequence, nt: nucleotide.

GyrA S83L, D87N and ParC S80I were strongly and significantly associated with resistance to fluoroquinolones. Triple mutations in *gyrA* (S83L and D87N/Y/G) and *parC* (S80I/R or E84K) were detected in 88% of the ciproR-*E. coli* and confer resistance to all tested fluoroquinolones. Two isolates contained a fourth mutation (GyrA S83L and D87N, ParC S80I and E84G) and one isolate additionally contained the *qnrS1* gene that showed MIC>32 mg/l for all fluoroquinolones. Outside of the QRDR in *gyrA* and *parC*, other mutations were detected in *gyrA*, *parC*, *gyrB*, *parE*, *acrB*, *acrR*, *marR*, *rpoB*, *soxR* and *soxS*, yet, were not positively associated with fluoroquinolone resistance (Figure 4). No mutations were detected in *marA*.

### 3.4. Genetic context of ESBL genes and PMQR in ESBL-producing and ciprofloxacin-resistant *Escherichia coli*

**Figure 5:**
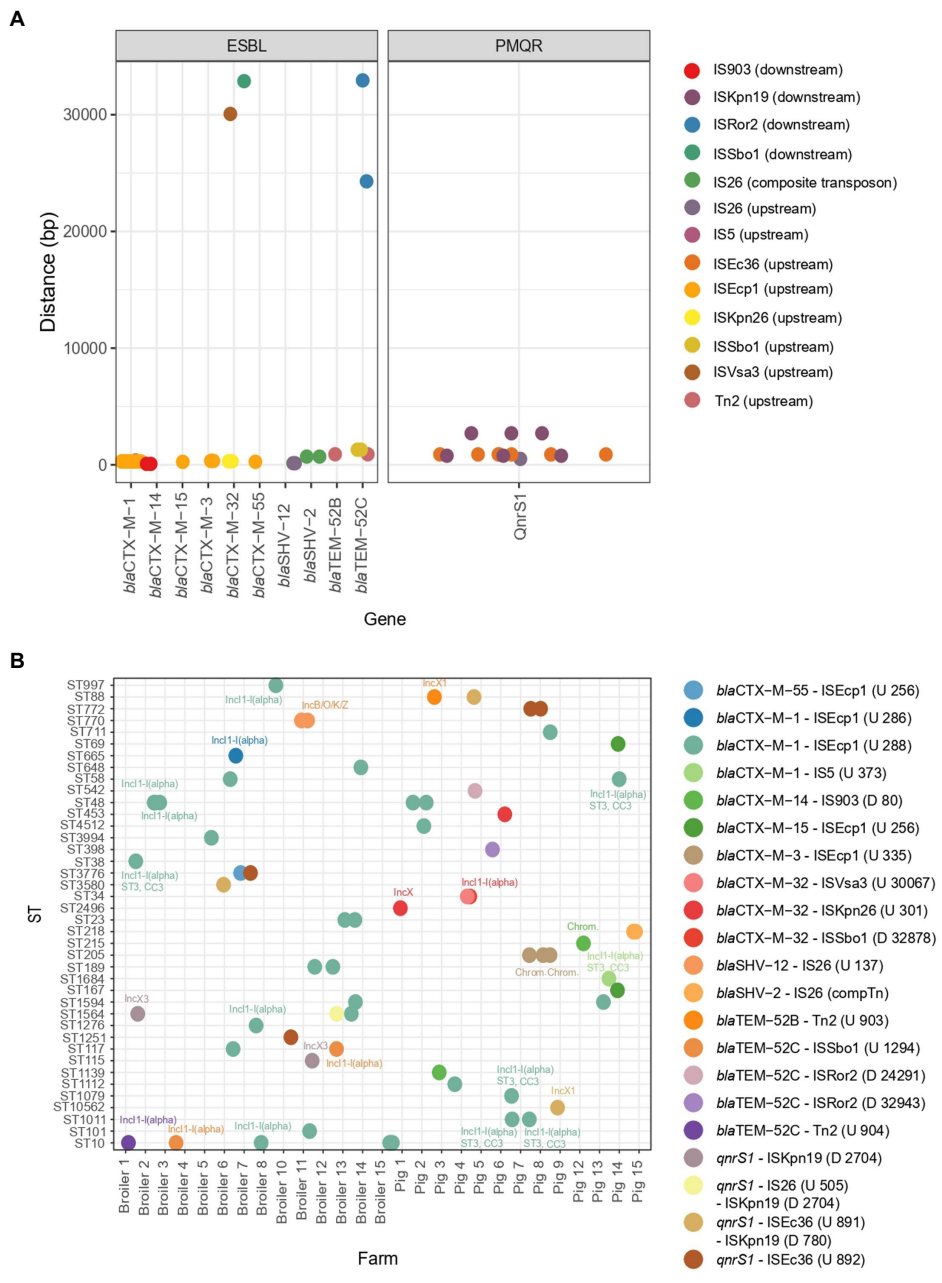
Mobile genetic elements and their association to ESBL genes and PMQR genes. **(A)** Distance of mobile genetic elements to the ESBL or PMQR gene. **(B)** The combination of the ESBL/PMQR gene with the closest mobile genetic element for every farm and ST element. The plasmid origins of replication are indicated in the figure. The distance and upstream (U)/downstream (D) location of the mobile genetic element are indicated in the figure legend.

The specific genetic context of ESBL and PMQR genes (closest MGE and, if possible, identification of plasmid origin or replication) could be identified for 66 isolates (Figure 5). MGEs tended to be present at a fixed distance from the resistance gene. ESBL gene *bla*_CTX-M-1_ was commonly found in association with ISEcp*1* upstream of the gene (*n* = 30) and was always detected on plasmids (Figure 5A). The plasmid IncI1-I(alpha) could be detected in 12 *bla*_CTX-M-1_-producing strains and, using pMLST, six of the IncI1-I(alpha) plasmids showed ST3, clonal complex 3. Evidently, this particular MGE circulates in six pig and 13 broiler farms amongst various *E. coli* genotypes, showcasing the remarkable distribution reach of this *bla*_CTX-M-1_ harboring plasmid (Figure 5B). Other resistance genes detected on a subset of the *bla*_CTX-M-1_-containing sequences are: *aadA5* (*n* = 2), *dfrA17* (*n* = 2), *mdtG* (*n* = 1), *mdtH* (*n* = 1), *mexA* (*n* = 1), *mexB* (*n* = 1), *qnrS1* (*n* = 1), *sul2* (*n* = 6) and *tetA* (*n* = 1), as well as virulence gene *cib* (*n* = 10). One porcine isolate harbored *bla*_CTX-M-1_ associated with IS*5* on an IncI1-I(alpha), ST3, CC3 plasmid. The IncI1-I(alpha) plasmid origin of replication could also be detected in association with other ESBL genes, such as *bla*_TEM-52C_ (*n* = 3) and *bla*_CTX-M-32_ (*n* = 1). The *bla*_SHV-12_ gene was detected on an IncN plasmid, without any association of IS elements in four broiler isolates from four different farms or in association with IS26 137 bp upstream of the *bla*_SHV-12_ gene on an IncB/O/K/Z plasmid in two isolates from a broiler farm. A composite transposon IS*26* surrounded the *bla*_SHV-2_ gene in isolates (*n* = 2) from a pig farm. Most ESBL genes were located on a plasmid. However, seven ESBL genes [*bla*_CTX-M-3_ (*n* = 3) associated with ISEcp*1*, *bla*_CTX-M-14_ associated with IS903 (*n* = 1), *bla*_CTX-M-15_ (*n* = 1) and *bla*_CTX-M-32_ (*n* = 2)] were predicted to be located on the chromosome. Different IS elements/transposons flanked the *bla*_CTX-M-32_ gene (upstream ISKpn26 (*n* = 2) on an IncX plasmid (*n* = 1) or downstream ISSbo1 on an IncI1-I(alpha) plasmid (*n* = 1) or upstream ISVas3 (*n* = 1)) and the *bla*_TEM-52C_ [upstream ISSbo1 (*n* = 2), upstream Tn2 (*n* = 1), downstream ISRor2 (*n* = 2)] in different isolates. The *bla*_TEM-52B_ gene was flanked by Tn2 in one porcine isolate and was located on an IncX1 plasmid. Co-localization of QnrS1 with *bla*_CTX-M-15_ (*n* = 1) or *bla*_CTX-M-55_ (*n* = 1) on a predicted plasmid contig was detected (Supplementary Figure 4). In 14 out of 75 isolates (18.7%), co-localization of virulence factor colicin Ib (*cib* gene, polypeptide toxins against *E. coli* and closely related bacteria) with an ESBL gene was detected.

The PMQR gene *qnrS1* was flanked by downstream ISKnp*19* (*n* = 6) and upstream either by ISEc*36* (*n* = 7) or by IS*26* (*n* = 1). For one porcine isolate, the plasmid replicon could be identified as IncX1 harbouring *bla*_TEM-1B_. For two broiler isolates from two different farms, QnrS1 could be located on an IncX3 plasmid (Figure 5B). QnrB19 was found to be located on a Col(pHAD) plasmid (*n* = 2); however, no IS elements flanking the gene could be identified. Also, no flanking MGEs could be identified for *oqxAB* genes.

### 3.5. Typing and possible transmission events of resistant *Escherichia coli* within and between farms

**Figure 6:**
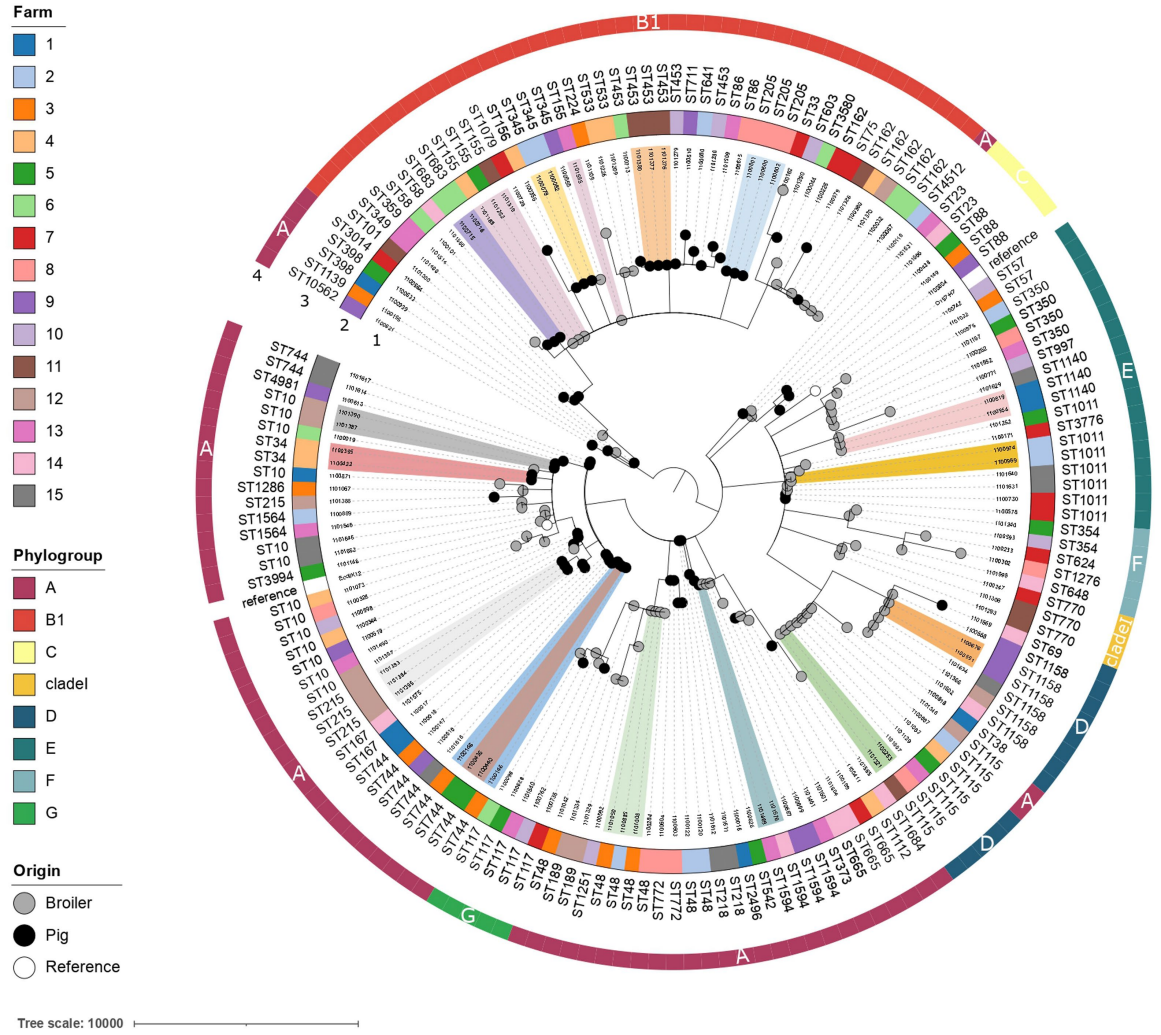
Phylogenetic tree of ciprofloxacin-resistant and ESBL-producing *E. coli* from broilers and pigs. The minimum spanning tree is distance-based and was generated by iTOL using cgMLST profile data (3,012 loci). Colored clusters indicate genetically related isolates with ≤10 allelic differences from different broilers/pigs. The isolate IDs are shown in the first ring. The farm is indicated in colored strips in the second ring. Achtmann ST and phylogroups are indicated in rings three and four, respectively. The origin of the isolate is indicated with black (pig), grey (broiler) or white (*E. coli* K12 and *E. coli* O157-H7 reference strains) nodes.

A highly diverse population of *E. coli* was isolated from broiler and pig farms (Figure 6). Overall, 63 different *E. coli* STs were detected with ST10 being the most abundant (13 out of 161 isolates, 8.1%). Phylogroup A was most common in ESBL-*E. coli* from broilers (47.6%) and pigs (57.1%) and in ciproR-*E. coli* from pigs (53.8%), where phylogroup B1 was most common among ciproR-*E. coli* from broilers (31.1%). Phylogroup A was most common among ESBL-*E. coli* from pigs (57.1%) and broilers (47.6%), and ciproR-*E. coli* from pigs (53.8%), while B1 was most common among ciproR-*E. coli* from broilers (31.1%). The number of virulence genes in phylogroups A and B1 was lower compared to phylogroups D and G (Supplementary Figure 5). FimH54 was the most common among ESBL-*E. coli* from broilers (16.7%) and pigs (40.0%) and ciproR-*E. coli* from pigs (41.0%), and fimH32 was most common among ciproR-*E. coli* from broilers (22.2%). With 85 different serotypes among 161 isolates, serotypes were widely diverse.

To determine the genetic relatedness of the isolates, a study specific cgMLST scheme with 3,012 loci was developed. Genetically linked bacterial clusters, with a maximal difference of 10 alleles among them (Schürch et al., 2018; Van Hoek et al., 2020), were identified on several pig (*n* = 8) and broiler farms (*n* = 3) (ST10, ST34, ST205, ST215, ST345, ST453, ST683, ST744, ST1011, ST1140, ST1158). Moreover, the presence of genetically similar resistant bacteria was detected between different broiler farms (*n* = 5) (ST115, ST48, ST155). These results suggest either transmission or a common reservoir between broiler farms. Transmission of *E. coli* ST1594 has likely occurred between a broiler farm and a pig farm as an allelic difference of 3 loci was shown between the two isolates (Figure 6).

### 3.6. Pathotypes detected in Belgian farm animals: ESBL-producing and ciprofloxacin-resistant ETEC and ESBL-producing EPEC

Most of the *E. coli* isolates were non-pathogenic. However, 12 pathogenic *E. coli* (7.45%) were detected in five pig farms and two broiler farms. ESBL-producing enterotoxigenic *E. coli* (ETEC) were detected in pig farms six (*n* = 2; phylogroup B1, CTX-M-32-producing) and 15 (*n* = 2 from the same pig; phylogroup A; SHV-2-producing) and ciprofloxacin-resistant ETEC were detected in pig farms eight (*n* = 2, ST772, phylogroup A, FimH54) and nine (*n* = 1, ST10, phylogroup A, FimH54). Enterotoxins *sta* and *stb* were present in 4 ETEC strains, *sta* was present in one ETEC strain and *stb* was present in two ETEC strains. The *stb*-containing contigs of the ETEC strains from pig farm 15 also contained the *astA* gene encoding the heat-stable enterotoxin (EAST1) and IS100, an IS21 family insertion element.

ESBL-producing enteropathogenic *E. coli* (EPEC) were detected in pig farm two (*n* = 2) and broiler farms four (*n* = 1) and 12 (*n* = 2). All EPEC strains were atypical because of the lack of bundle-forming pili (BFP). All EPEC strains were fimH54 belonging to phylogroup A; two were ST48 and CTX-M-1-producing strains, one was ST10 and TEM-52C producing strain and two were ST189 and CTX-M-1-producing strain. The latter two contained the IS256 composite transposon to mobilize the cassette of pathogenic virulence genes (*eae*, *espA*, *espB*, *espF*, *astA*, *tir*).

## 4. Discussion

The study showed that livestock is a reservoir for a large variety of antimicrobial resistance genes, virulence genes and plasmids. More than one type of ESBL gene was detected in most farms and *E. coli* belonging to a variety of STs was found in Belgian broilers and pigs.

The large collection of STs and serotypes of commensal *E. coli* in animals was described before (Ahmed et al., 2017; Duggett et al., 2020; Kaspersen et al., 2020; Massella et al., 2020; Zingali et al., 2020; Leekitcharoenphon et al., 2021). However, the pandemic multidrug-resistant clone ST131 commonly associated with human infections was not detected and *bla*_CTX M-15_ was rarely found [*n* = 3 from two pig farms (ST4981, ST69, ST167)]. *Escherichia coli* ST131 was also not detected in pig farms in Switzerland during a longitudinal study (Moor et al., 2021). The spread of *bla*_CTX-M-15_ in human-associated *E. coli* is globally linked to IncFII plasmids in ST131 (Rozwandowicz et al., 2018). IncFII plasmids were commonly detected (27.7% of the isolates) in this study but could not be linked to *bla*_CTX-M-15_ or ST131. Instead, CTX-M-1 predominates in *E. coli* from food-producing animals and food in Europe (Ceccarelli et al., 2019; Duggett et al., 2020). We found that the most common ESBL genes were *bla*_CTX-M-1_ and *bla*_SHV-12_ and ST10 was the most abundant sequence type. This is in line with other reports (Smet et al., 2008; Ahmed et al., 2017; Reid et al., 2017; van Damme et al., 2017; Ceccarelli et al., 2019; Stubberfield et al., 2019; Duggett et al., 2020; Zingali et al., 2020; Leekitcharoenphon et al., 2021; Moor et al., 2021). ST10 has been found in both humans, animals, retail meat and the environment (Oteo et al., 2009; Leverstein-van Hall et al., 2011; Toval et al., 2014; Manges et al., 2015; Reid et al., 2017), is associated with ESBL production (Oteo et al., 2009; Moor et al., 2021), and has been reported as an emerging extra-intestinal pathogen in humans, pigs and broilers (Riley, 2014; Manges and Johnson, 2015; Bojesen et al., 2022). The results from our study combined with published data confirm that ST10 is a potential dominant clonal group of commensal *E. coli* in food-producing animals globally. Other high-risk lineages (ST69, ST117, ST23, ST58, ST648, ST744) of *E. coli* were identified among our isolates. A total of 12 (7.45%) pathogenic *E. coli* strains were detected (ETEC and atypical EPEC), one ST10 TEM-52C-producing strain and two ST189 CTX-M-1-producing strains which contained an IS256 composite transposon to mobilize the cassette of pathogenic virulence genes (*eae*, *espA*, *espB*, *espF*, *astA*, *tir*). These composite transposons can move as a single unit to move these pathogenic virulence genes and disseminate them among bacteria.

The spread of ESBL genes is highly linked to epidemic and highly transmissible plasmids (Rozwandowicz et al., 2018; Kurittu et al., 2021). Most ESBL genes were predicted to be located on plasmids (91%) and were in the proximity of an IS element or transposon that was usually located at a fixed distance from the ESBL gene. The *bla*_CTX-M-1_ gene was often associated with ISEcp*1* and IncI1-I(alpha)-ST3 in several broiler and pig farms, as described before (de Been et al., 2014; Zurfluh et al., 2014; Partridge et al., 2018; Rozwandowicz et al., 2018; Ceccarelli et al., 2019; Bernreiter-hofer et al., 2021). ISEcp*1* is known to be associated with ESBL genes. Genes downstream of this IS element can be mobilized through transposition (including chromosomal integration) and are able to enhance ESBL gene expression under its own promotor (Poirel et al., 2003; Ceccarelli et al., 2019; Massella et al., 2020). In our study, the IncI1-I(alpha) plasmid was also found to carry other ESBL genes (*bla*_CTX-M-32_ and *bla*_TEM-52C_). These results indicate that the IncI1-I(alpha) plasmid is a major plasmid type contributing to the spread of ESBLs in Belgian farms. Other ESBL-plasmid origin-of-replication combinations were: bla_SHV-12_ on an IncN plasmid or IncB/O/K/Z plasmid, bla_CTX-M-32_ on an IncX plasmid and bla_TEM-52B_ on an IncX1 plasmid. QnrS1 seems to be flanked by different IS elements and was located on IncX1 in a pig farm or IncX3 plasmids in two broiler farms. IncX plasmids were described as widely distributed and to be associated with fluoroquinolone resistance (Dobiasova and Dolejska, 2016). The presence of QnrS1 on IncX1 or IncX3 plasmids was shown before in Germany’s pork and beef production chain (Juraschek et al., 2021). QnrB19 could be located on a Col(pHAD) plasmid in two isolates in our study, which was also the case in *Salmonella* spp. from poultry in Nigeria (Jibril et al., 2021).

Co-localization of ESBL genes with virulence factor *cib* was detected in 14/75 isolates (18.7%) and co-localization with other resistance genes (such as *aadA* genes, *dfrA* genes, *aph(3′)-Id*, *aph(6)-Id*, *bla*_TEM-1B_, *cmlA1*, *sul* genes, *tetA*, *and qnrS*) was detected. PMQR and ESBL genes localized on the same presumed plasmid contig [*qnrS1* with *bla*_CTX-M-15_ (*n* = 1) or *bla*_CTX-M-55_ (*n* = 1)] is concerning. Plasmids co-harboring multiple resistance determinants to critically important antibiotics for human medicine limit treatment options for severe infections and are a threat to public health.

PMQR genes were found in a remarkably low number of isolates and play a limited role in the occurrence of ciproR-*E. coli* in Belgian farms. Ciprofloxacin resistance was caused by mutations in the QRDR region of *gyrA* and *parC* in all ciproR*-E. coli*, of which most showed triple mutations (GyrA S83L and D87N and ParC S80I) significantly associated with high-level fluoroquinolone resistance. In contrast, QnrS1 or QnrB19 alone leads to low-level resistance to enrofloxacin and moxifloxacin and a sensitive/intermediate phenotype for ciprofloxacin and levofloxacin. Despite strong negative correlations between the presence of *qnr* genes and *gyrA* mutations shown previously and the hypothesis that Qnr proteins have a protective effect on quinolone targets (Kaspersen et al., 2020), the presence of QnrS1 combined with GyrA S83L amino acid change was almost always detected in our study. Only two porcine ciproR-*E. coli* isolates did not contain any mutations in the QRDR of *gyrA* and *parC*, instead harbored two PMQR (OqxAB and QnrS1). Although PMQR mechanisms provide low-level resistance (Jacoby et al., 2015), the combination of OqxAB and QnrS1 was sufficient to result in fluoroquinolone resistance above breakpoint.

Pig isolates showed a higher mean number of resistance genes, especially for porcine ciproR-*E. coli*, which could reflect the higher use of antibiotics in pigs compared to broilers (FOD Volksgezondheid Veiligheid van de Voedselketen en Leefmilieu et al., 2021). In contrast, virulence genes were more abundantly present in broiler isolates. Most virulence genes were involved in adherence and invasion (most prevalent virulence genes were *iss, gad, lpfA*), which can contribute to successful colonization and enhanced survival in the gut and the environment (Projahn et al., 2018). Also, the presence of ExPEC-associated virulence factors (such as *astA*, *iss*, *iha*, and *iroN*) is an indication that these commensal *E. coli* in Belgian farms may have pathogenic potential (Mo et al., 2016). Phylogroups A and B1 were the most common and are associated with commensal phenotypes (Johnson and Stell, 2000). In line with this, phylogroups A and B1 carried a lower number of virulence genes compared to phylogroups D and G. However, the pathogenic *E. coli* (ETEC and EPEC) detected in this study belonged to phylogroups A and B1 showing that these phylogroups also have the potential to cause extraintestinal infections.

We identified multiple genetically related clones in different animals of the same farm and of distinct farms. The presence of clonally-related bacteria in different poultry farms suggests a common reservoir or transmission of resistant bacteria. The vertical spread of resistant bacteria from the top to the bottom of the broiler production pyramid (Dierikx et al., 2013; Zurfluh et al., 2014) and resistant *E. coli* residing in the farm environment (Blaak et al., 2015) were previously identified as important transmission routes of resistant bacteria. The diverse profiles of resistance genes, virulence genes and plasmid profiles reflect complex epidemiology. In addition, the detection of plasmid replicons with associated IS elements and ESBL/PMQR genes in different farms and among several STs (such as IncI1-I(alpha) and IncX3) underline that plasmid transmission could be another important contributor to the transmission of resistance.

Our data show the complex epidemiology of ESBL-production and ciprofloxacin resistance in *E. coli* from livestock, suggesting the spread of these resistances involves both dissemination of resistant clones and horizontal transmission of plasmids. This emphasizes how critical it is to curtail the unnecessary use of antibiotics across all levels of the livestock production chain to preserve antibiotic effectiveness. Additionally, further research into plasmid involvement should include sequencing over longer reads to better understand its circulation on farms. The study supports that commensal *E. coli* in livestock should be monitored using WGS. Although not all resistance genes could be associated with MGEs or plasmids and we only sequenced a sub-selection of the resistant strains per farm, we gained valuable information on the genetic characteristics of ESBL-*E. coli* and ciproR-*E. coli* and the transmission of clones and resistance genes in Belgian farms using genomic data.

## 5. Conclusion

Our research uncovers a multifaceted landscape of ESBL production and ciprofloxacin resistance in Belgian farms. The complex epidemiology with diverse combinations of ESBL genes, ST types, antibiotic resistance and virulence profiles makes it difficult to translate these findings to the impact on human health. Nevertheless, WGS provides detailed information and should be utilized to properly track this complex situation which poses an urgent challenge for preventing the spread of antimicrobial resistance in the broiler and pig production chain.

## Data availability statement

The datasets presented in this study can be found in online repositories. The names of the repository/repositories and accession number(s) can be found in the article/Supplementary material.

## Ethics statement

Ethical review and approval was not required for the animal study because the procedure to collect fresh fecal droppings is considered to cause no discomfort, and animals were neither handled nor sacrificed during the study (EC Directive 2010/63). Written informed consent was obtained from the owners for the participation of their animals in this study.

## I-4-1-Health Study Group

Lieke van Alphen, Maastricht University Medical Center +, Maastricht, the Netherlands; Nicole van den Braak, Avans University of Applied Sciences, Breda, the Netherlands; Caroline Broucke, Agency for Care and Health, Brussels, Belgiu; Anton Buiting, Elisabeth-TweeSteden Ziekenhuis, Tilburg, the Netherlands; Liselotte Coorevits, Ghent University Hospital, Ghent, Belgium; Sara Dequeker, Agency for Care and Health, Brussels, Belgium; Jeroen Dewulf, Ghent University, Ghent, Belgium; Wouter Dhaeze, Agency for Care and Health, Brussels, Belgium; Bram Diederen, ZorgSaam Hospital, Terneuzen, the Netherlands; Helen Ewalts, GGD Hart voor Brabant, Tilburg, the Netherlands; Herman Goossens, University of Antwerp, Antwerp, Belgium and Antwerp University Hospital, Antwerp, Belgium; Inge Gyssens, Hasselt University, Hasselt, Belgium; Casper den Heijer, GGD Zuid-Limburg, Heerlen, the Netherlands; Christian Hoebe, Maastricht University Medical Center+, Maastricht, the Netherlands and GGD Zuid-Limburg, Heerlen, the Netherlands; Casper Jamin, Maastricht University Medical Center+, Maastricht, the Netherlands; Patricia Jansingh, GGD Limburg Noord, Venlo, the Netherlands; Jan Kluytmans, Amphia Hospital, Breda, the Netherlands and University Medical Center Utrecht, Utrecht University, Utrecht, the Netherlands; Marjolein Kluytmans–van den Bergh, Amphia Hospital, Breda, the Netherlands and University Medical Center Utrecht, Utrecht University, Utrecht, the Netherlands; Stefanie van Koeveringe, Antwerp University Hospital, Antwerp, Belgium; Sien De Koster, University of Antwerp, Antwerp, Belgium; Christine Lammens, University of Antwerp, Antwerp, Belgium; Isabel Leroux, Ghent University Hospital, Ghent, Belgium; Hanna Masson, Agency for Care and Health, Brussel, Belgium; Ellen Nieuwkoop, Elisabeth-TweeSteden Ziekenhuis, Tilburg, the Netherlands; Anita van Oosten, Admiraal de Ruyter Hospital, Goes, the Netherlands; Natascha Perales Selva, Antwerp University Hospital, Antwerp, Belgium; Merel Postma, Ghent University, Ghent, Belgium; Stijn Raven, GGD West-Brabant, Breda, the Netherlands; Paul Savelkoul, Maastricht University Medical Center+, Maastricht, the Netherlands; Annette Schuermans; University Hospitals Leuven, Leuven, Belgium; Nathalie Sleeckx, Proefbedrijf Pluimveehouderij VZW, Geel, Belgium; Krista van der Slikke, GGD Zeeland, Goes, the Netherlands; Arjan Stegeman, Utrecht University, Utrecht, the Netherlands; Tijs Tobias, Utrecht University, Utrecht, the Netherlands; Paulien Tolsma, GGD Brabant Zuid-Oost, Hertogenbosch, the Netherlands; Jacobien Veenemans, Admiraal de Ruyter Hospital, Goes, the Netherlands; Dewi van der Vegt, PAMM Laboratory for pathology and medical microbiology, Veldhoven, the Netherlands; Martine Verelst, University Hospitals Leuven, Leuven, Belgium; Carlo Verhulst, Amphia Hospital, Breda, the Netherlands; Pascal De Waegemaeker, Ghent University Hospital, Ghent, Belgium; Veronica Weterings, Amphia Hospital, Breda, the Netherlands and Radboud University Medical Center, Nijmegen, the Netherlands; Clementine Wijkmans; GGD Hart voor Brabant, Tilburg, the Netherlands; Patricia Willemse–Smits, Elkerliek Ziekenhuis, Geldrop, the Netherlands; Ina Willemsen, Amphia Hospital, Breda, the Netherlands.

## Author contributions

SK: original draft writing. MR, BX, CL, DC, KB, KM, JD, MK, JK, and HG: review and editing. SK and MR: data collection. SK, BX, DC, KB, and KM: data analysis. HG and JK: funding acquisition. MB, JK, and HG: project administration. HG, CL, MK, and JK: supervision. SK and MK: data curation. All authors contributed to the article and approved the submitted version.

## Funding

The i-4-1-Health project was financed by the Interreg V Flanders-The Netherlands program, the cross-border cooperation program with financial support from the European Regional Development Fund (ERDF) (0215). Additional financial support was received from the Dutch Ministry of Health, Welfare and Sport (325911), the Dutch Ministry of Economic Affairs (DGNR-RRE/14191181), the Province of Noord-Brabant (PROJ-00715/PROJ-01018/PROJ-00758), the Belgian Department of Agriculture and Fisheries (no reference), the Province of Antwerp (1564470690117/1564470610014) and the Province of East-Flanders (E01/subsidie/VLNL/i-4-1-Health). The authors are free to publish the results from the project without interference from the funding bodies. Selective and non-selective agar plates, Etests and Vitek2 AST cards were provided by bioMérieux; FecalSwabs and tryptic soy broths were provided by Copan. The authors were free to publish the results from the project without interference by bioMérieux or Copan.

## Conflict of interest

The authors declare that the research was conducted in the absence of any commercial or financial relationships that could be construed as a potential conflict of interest.

## Publisher’s note

All claims expressed in this article are solely those of the authors and do not necessarily represent those of their affiliated organizations, or those of the publisher, the editors and the reviewers. Any product that may be evaluated in this article, or claim that may be made by its manufacturer, is not guaranteed or endorsed by the publisher.

## Footnotes

^1^ https://github.com/FelixKrueger/TrimGalore
