# Supplementary material for: Genetic characterization of ESBL-producing and ciprofloxacin-resistant Escherichia coli from Belgian broilers and pigs
Source: Front Microbiol. 2023 Apr 6;14:1150470. doi: 10.3389/fmicb.2023.1150470 (PMC10116946; doi:10.3389/fmicb.2023.1150470)
Supplement: Supplementary file 3 [file Data_Sheet_1.docx]

Supplementary Material

Genetic characterization of ESBL-producing and ciprofloxacin-resistant *Escherichia coli* from Belgian broilers and pigs

**Sien De Koster, Moniek Ringenier, Basil Britto Xavier, Christine Lammens, Dieter De Coninck, Katrien De Bruyne, Klaas Mensaert, Marjolein Kluytmans-van den Bergh, Jan Kluytmans, Jeroen Dewulf, Herman Goossens^*^ , on behalf of the i-4-1-Health Study Group**

*** Correspondence:** Herman Goossens: herman.goossens@uza.be

# Supplementary Data

## Supplementary Figures

##

## Supplementary Figure 1: Number of virulence genes and rank 1 resistance genes (current threats). The size of the bubbles represents the number of isolates, colors indicate origin of the isolates (pig: dark color, broiler: light color) and resistance mechanism (ESBL-producing *E. coli*: green, ciprofloxacin-resistant *E. coli*: blue).

**Supplementary Figure 2**: Heatmap of presence (dark green) and absence (light green) of plasmid origin of replications in ESBL-producing and ciprofloxacin-resistant *E. coli* isolated from broilers and pigs. Each row relates to an isolate and each column represents a plasmid origin of replication. White vertical lines separate isolates from the same farm.

**Supplementary Figure 3**: Number of virulence genes according to the role of the gene in pathogenesis and life-style in ESBL-*E. coli* and CiproR-*E. coli* isolated from broilers and pigs. Statistically significant differences are indicated according to the level of significance: * (p<0.05), ** (p<0.01), *** (p<0.001) (ANOVA with TukeyHSD or Games-Howell post-hoc tests).

**Supplementary Figure 4**: Genetic context of ESBL gene and PMQR co-localized in the same genetic region. Blue bars represent mobile genetic elements, grey bars are hypothetical proteins and red bars are ESBL genes or PMQR.

**Supplementary Figure 5:** Number of virulence genes for each phylogroup. Statistically significant differences are indicated according to the level of significance: * (p<0.05), ** (p<0.01) (ANOVA with Games-Howell post-hoc test).
